# Open ascending aorta replacement combined with fenestrated total aortic arch stenting

**DOI:** 10.1016/j.xjtc.2025.03.002

**Published:** 2025-03-08

**Authors:** Xiantao Ma, Zhangqiang Zhu, Yi Feng, Chenhe Li, Ningxin Hou, Cai Cheng

**Affiliations:** a Division of Cardiothoracic and Vascular Surgery, Tongji Hospital, Tongji Medical College, Huazhong University of Science and Technology, Wuhan, China; b Department of Cardiovascular Surgery, The Second Affiliated Hospital of Zhejiang University School of Medicine, Hangzhou, China

**Keywords:** type A aortic dissection, TEVAR, fenestration technique, endovascular treatment, hybrid technique

## Abstract

**Objective:**

The aim of this retrospective single-center study was to report a novel hybrid technique of replacement of the ascending aorta and implantation of fenestrated stent-graft of the entire aortic arch for Stanford type A aortic dissection and to analyze clinical experience and outcomes.

**Methods:**

From January 2019 to January 2023 in Tongji hospital, 31 cases (26 men and 5 women, mean age 56.06 ± 10.34 years) with Stanford type A aortic dissection underwent open ascending aorta replacement combined with total endovascular arch repair. All patients underwent ascending aorta replacement without hypothermia or circulatory arrest. Arch intervention was performed with self-modified stent-grafts to preserve the aortic arch native branches.

**Result:**

The surgical technical success rate was 100% in all patients. One (3.22%) patient died after surgery due to cerebral hemorrhage. Five (16.13%) patients with preoperative renal insufficiency required hemodialysis. Six (19.36%) patients were on mechanical ventilation for more than 48 hours. One patient was found to have an endoleak (Type IV). There were 25 (83.33%) patients who underwent follow-up with a median follow-up time of 14.00 months (range, 6.50-28.50 months). Two (8.00%) of them died (1 of infectious shock and the other of respiratory arrest) and 2 (8.00%) underwent aortic reoperation.

**Conclusions:**

Single-stage open ascending aortic replacement combined with the total aortic endovascular arch intervention may provide satisfactory early outcomes in Stanford type A aortic dissection. This strategy may be valuable for a subgroup of patients deemed inappropriate candidates for open classic full arch repair.

**Figure:**
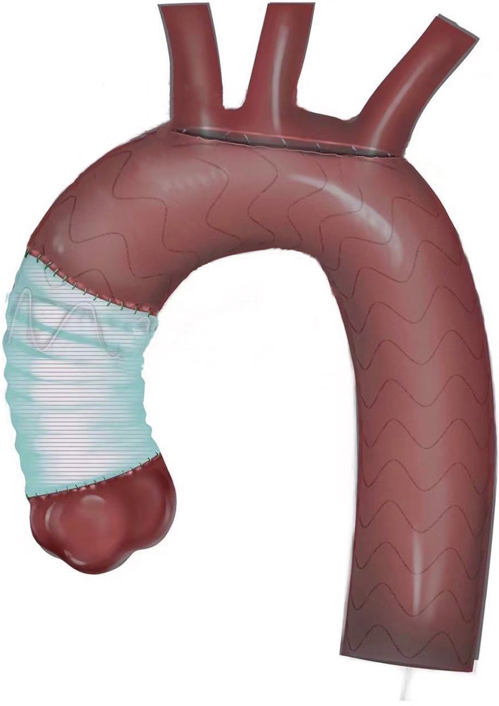
Morphology of ascending aorta replacement combined with fenestrated stenting.

**Central Message**

For type A aortic dissection, ascending aorta replacement combined with fenestrated stent-graft covering the totality of the aortic arch provides early positive outcomes in selected patients.

**Perspective**

Hybrid technology is used for ascending aortic replacement and total arch repair as a viable option for some patients with type A aortic dissection. It offers a promising alternative for patients who are not suitable for traditional total arch surgery, and early results show that it can safely and effectively treat this life-threatening condition.

Acute type A aortic dissection (TAAD), characterized by an intimal tear creating a life-threatening false lumen involving the ascending aorta and arch, traditionally requires open surgical repair via median sternotomy with cardiopulmonary bypass (CPB) and hypothermic circulatory arrest (HCA).^1^^,^^2^ Although modern cerebral protection strategies have improved outcomes, total/hemiarch replacement remains highly invasive, particularly for elderly or comorbid patients, with substantial morbidity and resource demands.

Thoracic endovascular aortic repair (TEVAR), now first-line therapy for type B dissections, has inspired hybrid approaches combining ascending replacement with endovascular arch stabilization.^3^ However, existing hybrid techniques often necessitate HCA, prolonged operative times, and complex hemostasis management.^4^^,^^5^

To address these limitations, we developed a novel single-stage hybrid protocol: Open ascending replacement (without HCA) followed by total arch endovascular repair using intraoperatively modified fenestrated stent-grafts (SGs). This study evaluates early outcomes of this streamlined approach in selected patients with TAAD.

## Materials and Methods

### Patients

From January 2019 to January 2023 in Tongji Hospital, 31 patients with TAAD underwent ascending aorta replacement combined with total arch repair were treated using implantation of a self-modified fenestrated SG replacement. Patients were informed and signed consent forms preoperatively. Among these patients, a total of 26 patients were men and 5 patients were women, with age 56.06 ± 10.34 years. The patients' data were retrospectively reviewed and analyzed after obtaining Ethics Committee of Tongji Hospital, Tongji Medical College, Huazhong University of Science and Technology approval for the study (institutional review board document No. TJ-IRB20220124; January 17, 2022). Written informed consent was obtained from all participating patients or their legal guardians for both the surgical procedure and the publication of anonymized study data. Contrast-enhanced computed tomography angiography (CTA) was used to confirm the diagnosis of all patients before operation. The preoperative dates of all patients in our study are presented in Table 1.

**Table 1:** Patient characteristics

Variable	Result
Male	26 (83.87)
Age (y)	56.06 ± 10.34
Entry tear	
Ascending aorta	20 (64.52)
Descending aorta	5 (16.13)
No visible tears found	3 (9.68)
Multiple tears	3 (9.68)
History	
Hypertension	24 (77.42)
Coronary artery disease	2 (6.45)
Chronic renal failure/eGFR <50	3 (9.68)
Previous cardiovascular surgery	3 (9.68)
History of smoking	18 (58.06)
History of drinking alcohol	17 (54.84)

Values are presented as n (%) or mean ± SD. *eGFR*, Estimated glomerular filtration rate.

### Indications for the Current Approach

All patients were diagnosed by CTA at admission as having TAAD, the dissection flap did not extend into or compromise the true lumen of the aortic arch branches, the ascending aorta replacement could provide enough landing zone for secondary arch endovascular anchorage, and there was no rupture on the side of the greater curvature of the aortic arch when the lesion involved the aortic arch. Additionally, the patients did not have Marfan syndrome; had no severe calcification of the ascending aorta wall or multiple lacerations of the proximal aortic arch; did not present with acute cardiocerebrovascular accident, severe hepatorenal insufficiency, intestinal ischemia, or severe coagulopathy; and did not have an allergy to the contrast medium or graft metal.

### Description, Planning, and Sizing of Fenestrated SG Preparation

Preoperative CTA was used to determine the location of the primary tear, anatomical features of the aortic arch, and the location of the epiaortic vessels, as well as the distance of each target vessel's diameter, length, and spacing. The SG sizes used in this study were 24 to 32 mm and consisted of multiple self-expandable nitinol wire rings connected by a polyester vascular fabric felt that were modified and manufactured by Medtronic and Lifetech. Each patient's aortic configuration was taken into consideration when modifying the SG.

**Figure 1:**
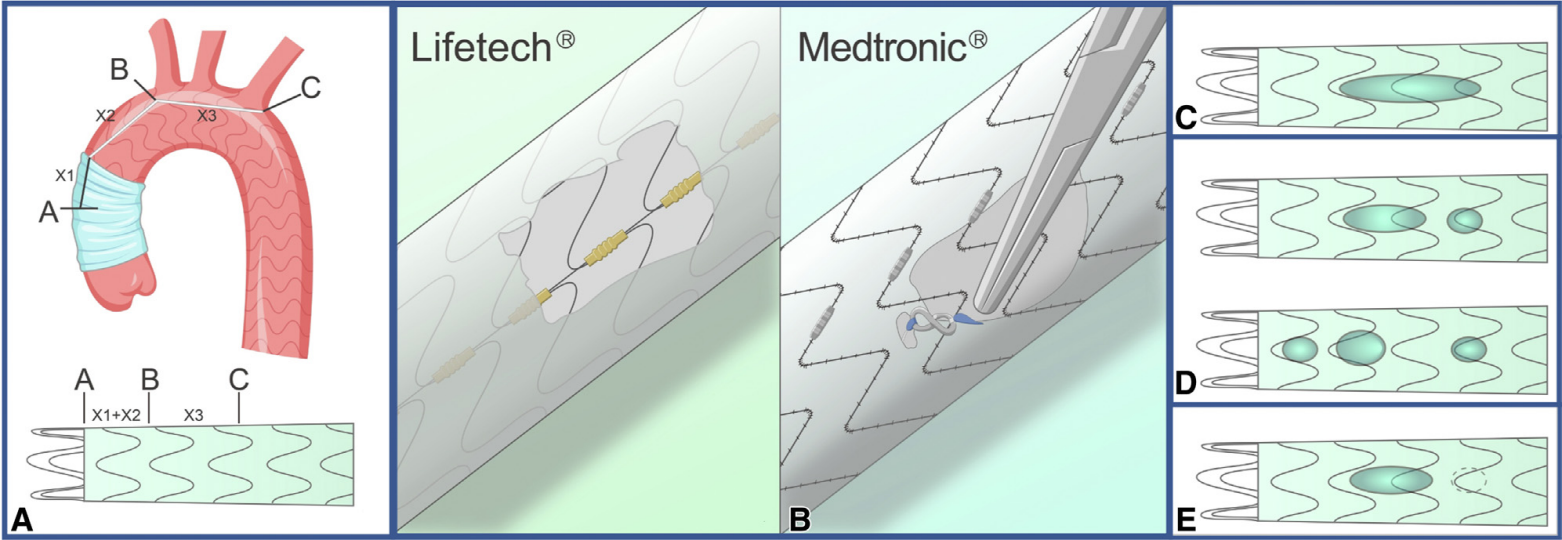
Schematic diagram. A, Designing window sizes for fenestrated total aortic arch stenting. B, Marker of the arch upper curve. C through E, Fenestration methods.

The SG modification was done on a back table. We measured the distance from the anastomosis site to the proximal point of the opening of the main trunk of the brachial artery (X2 mm), and from the proximal point of the opening of the main trunk of the brachial artery to the distal point of the opening of the left subclavian artery (X3 mm). According to the preoperative measurement results and position, the stent was placed at the front end of the stent beyond the anastomosis orifice by X1 = 10-15 mm. The distance between the initial position of the opening from the front end of the stent was AB = X1 + X2 mm, and the length of the fenestration was BC = X3 mm (Figure 1, *A*).

### Mark and Modification of the Fenestrated Side in the SG

We need to confirm the orientation of the SG and marker because the fenestration was shaped to lie on the upper curve of the aortic arch (Figure 1, *B*). There is a line of Lifetech SGs that are radio-opaque under radiographs, and the stent can be shaped on the side of the line. Although Medtronic's SGs lack markers, it is necessary to sew radio-opaque markers (coming from either the SG itself or the radiograph contrast tube) under radiograph on the edges of the fenestrated side of the SG to serve as a marker during the operation.

According to the measurements acquired from preoperative analysis, the position of the dissection and the distance from the 3 branches on the arch can be determined. The opening of the fenestration can then be flexibly selected:

• In vitro fenestration (single fenestration). If the dissection does not involve the aortic arch, 1 single window is opened (Figure 1, *C*).
• In vitro fenestration (double/triple fenestration). If the dissection involves the aortic arch, or the tear is on the side of the lesser curvature, to prevent the occurrence of leakage we can use double or triple windows for aortic arch pathologies (Figure 1, *D*).
• In vitro fenestration plus in situ fenestration. If the dissection is very close to a branch, we can use in situ fenestration (Figure 1, *E*).

### Ascending Aorta Replacement and Arch SG Implantation

We performed all procedures in a single stage in a hybrid operating theater outfitted with a C-arm fluoroscopy system (AXIOM Artis FA; Siemens). After successful general anesthesia, all patients were placed supine and we exposed the right axillary artery and right femoral artery for cannulation. CPB was achieved after a full median sternotomy and heparinization via 2-stage venous cannula through the right atrial appendage and 2 arterial return cannulas through the right axillary and femoral arteries.

The ascending aorta was occluded before the takeoff of the brachiocephalic trunk. The aorta was opened longitudinally, and cardioplegia (custodiol-HTK or Del Nido cardioplegia) was perfused directly into the opening of the coronary artery for cardiac arrest and myocardial protection. After the cardiac arrest was satisfactory, the aortic root was examined to determine the extent of proximal intervention. In cases where the dissection tear extended beyond the crossclamp site, the distal anastomosis was reinforced with felt strips and biological glue, whereas intraoperative imaging guidance ensured coverage of distal lesions with SGs to secure anastomotic integrity.

**Figure 2:**
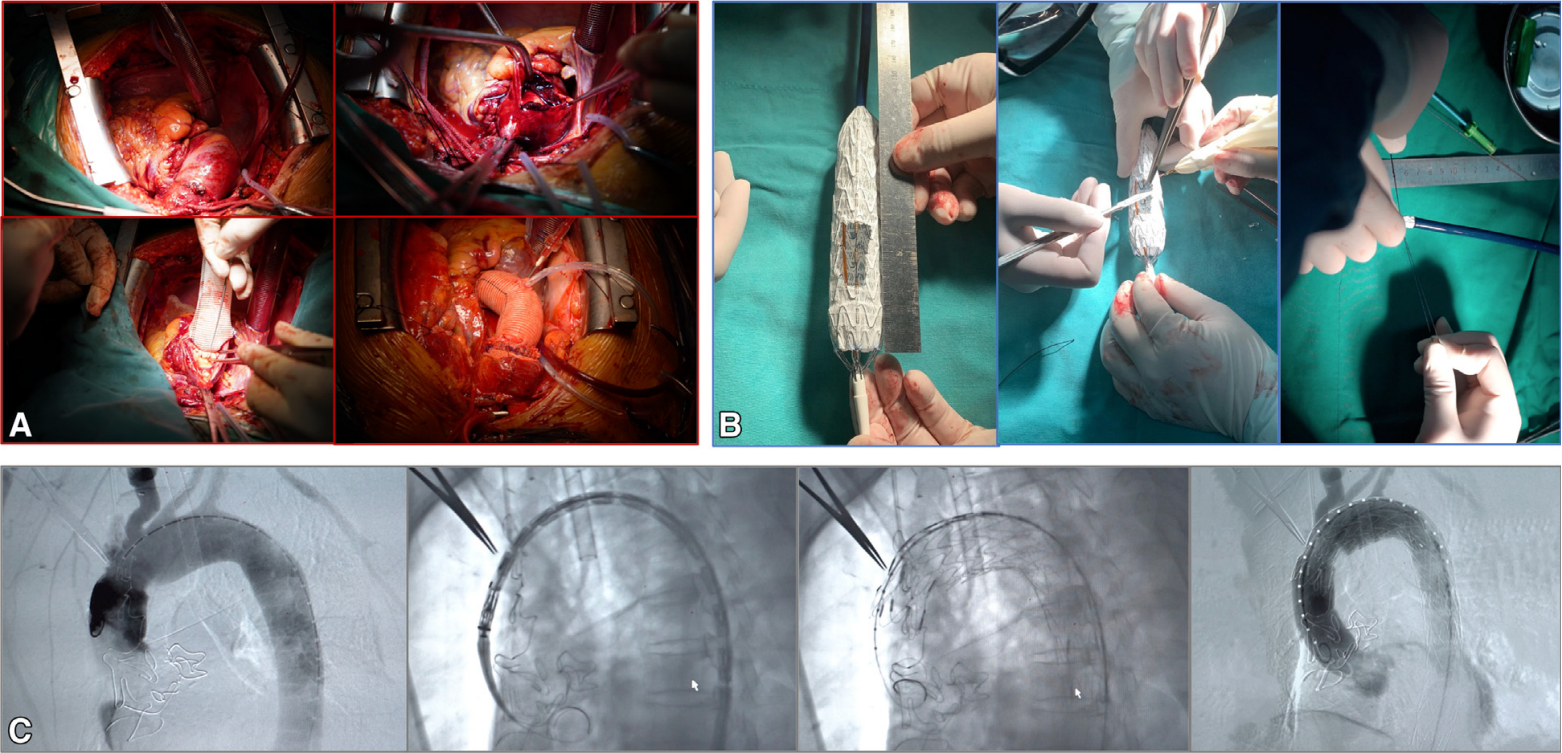
Ascending aorta replacement combined with total aortic arch fenestrated technique. A, Procedures for ascending aortic replacement. B, Based on preprocedural measurements, the opening was performed on the back table and the stent was put back into the sheath. C, Endovascular repair of thoracic aorta (the stent completely covered the entire aortic arch).

Then, the ascending aorta was replaced with a tube vascular graft (Terumo) of corresponding length and diameter by end-to-end anastomosis with 3-0 or 4-0 Prolene sutures (Ethicon). The proximal end is anastomosed initially, followed by the distal end. When the arterial wall tissue was weakened, the sandwich technique was utilized.^6^ If there was bleeding at the anastomosis site, a modified Cabrol shunt was utilized.^7^ After anastomosis and sufficient hemostasis, the aortic crossclamp was released and contractility was recovered and CPB support was discontinued (Figure 2, *A*).

The endovascular procedure was performed before sternal closure. A single wide opening fenestration was created from the brachiocephalic trunk to the left subclavian artery if the quality of the greater curvature of the aortic arch was high (Figure 2, *B*). The distal aortic anastomosis and the beginning and ending of the arch branches were marked with titanium clips or small cured clamps and the incision membrane was attached to the anterior thoracic incision before the SG implantation was performed. The right femoral artery was the access point for the stent delivery with a 6 Fr sheath. The guidewire and pigtail catheter (Cook Medical) were inserted. Aortography was performed and the image was saved for navigating display (Figure 2, *C*).

### Calculation Angle

During the digital subtraction angiography, the left anterior oblique range is 30° to 60°, and the head and foot position should be 0°. With the marker on the screen to accurately delineate the position of the aortic arch and branches, it is important to determine the profile of the branch of the aortic arch. The guide wire should be close to the greater curvature side of the aortic arch. When the delivery system enters from the femoral artery, the marking point of the opening position should be perpendicular to the ground; that is, at 6 o'clock. After entering the femoral artery, the delivery system maintains the original clock position without rotating. When the delivery system reaches the fenestration position of the aortic arch, it turns to the top of the arch; that is, the 12 o'clock position.

### Postoperative Follow-up and Statistical Methods

Follow-up will be done on an outpatient basis and/or by telephone, and patients will be asked to undergo a thoracoabdominal aortic CTA at 6 months. The data were presented in the following ways: categorical variables are presented as frequency and percentage values, whereas variables are shown as mean or median according to results from the Kolmogorov-Smirnov test, and SPSS version 20.0 (SPSS Inc) was used for data analysis.

## Results

### Patient Characteristics

The baseline characteristics of all patients are presented in Table 1. The mean age was 56.1 ± 10.3 years of whom 83.8% were men. There were 24 patients with hypertension, 2 patients with coronary artery disease, 3 patients with renal insufficiency, 3 patients with previous aortic surgeries, 18 patients with a history of smoking, and 17 patients with a history of alcohol drinking. Of these patients, the dissection involved the aortic arch but did not extend into the origins of the arch branch vessels (brachiocephalic, left common carotid, or left subclavian arteries) or compromise their true lumens.

### Operation Data

As summarized in Table 2, we performed a new simplified hybrid approach of ascending aorta replacement combined with total aortic arch fenestration. The total operative time was 7.05 ± 2.45 hours, CPB time was 2.69 ± 1.17 hours, the aortic crossclamp time was 1.23 ± 0.59 hours, and intraoperative erythrocytes were used at 6.00 U (range, 3.13-6.50 U). The patients stayed the intensive care unit for 3.00 days (range, 2.00-6.00 days) and were discharged 17.53 ± 9.94 days after surgery. The median number of stents used was 1.00 (range, 1.00-2.00) and the mean stent diameter was 29.68 ± 1.92 mm.

**Table 2:** Operative data

Variable	Result
Emergency operation	19 (61.29)
Methods of operation	
Ascending aorta replacement	23 (74.19)
Coronary artery bypass grafting	1 (3.26)
Bentall procedure	4 (12.90)
Wheat procedure	1 (3.26)
Cabrol procedure	1 (3.26)
David procedure	1 (3.26)
Operation time (min)	7.05 ± 2.45
No. of stent-grafts	1.00 (1.00-2.00)
Stent-graft diameter (mm)	29.68 ± 1.92
RBC transfusion (U)	6.00 (3.13-6.50)

Values are presented as n (%), median (interquartile range), or mean ± SD. *RBC*, Red blood cell.

### Early Outcomes and Follow-up Outcomes

**Figure 3:**
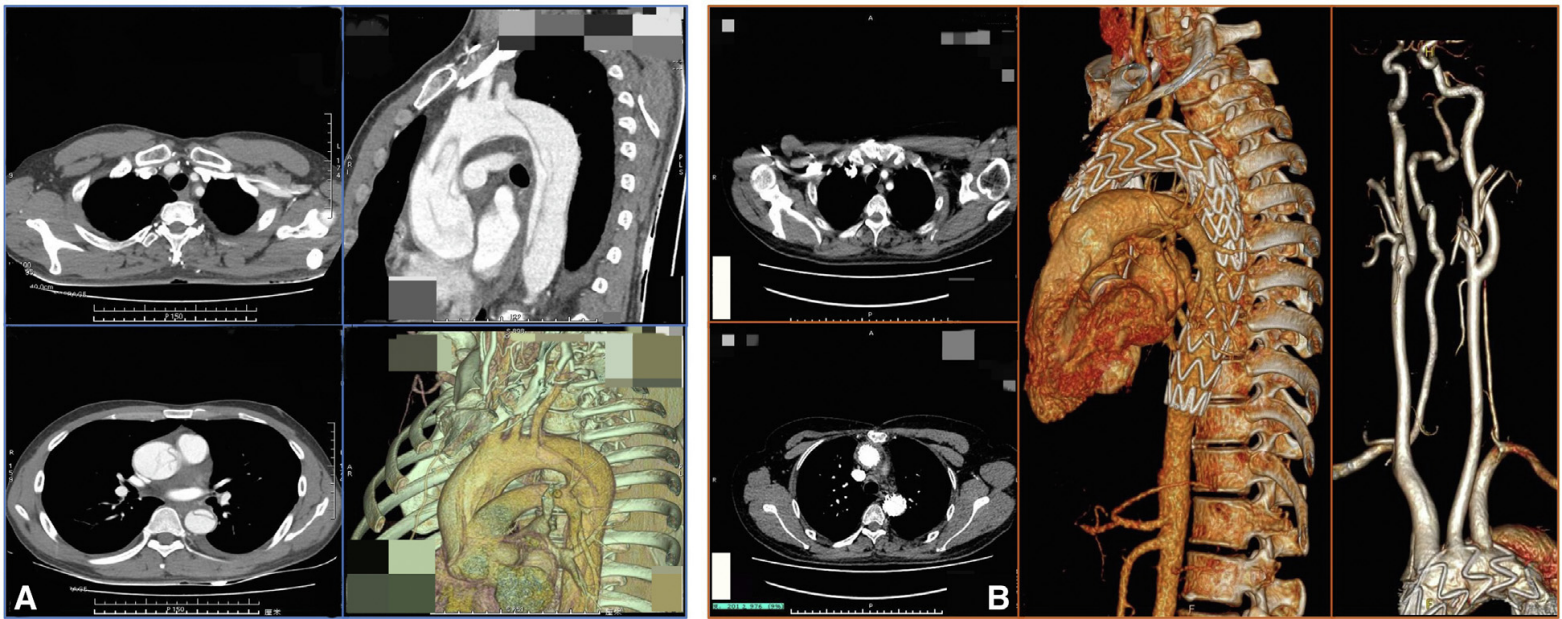
Images of computed tomography angiography (*CTA*). A, Preoperative CTA. B, Postoperative CTA.

As shown in Table 3, the surgical technical success rate was 100%. Successful surgical technique was defined as completion of aortic replacement, fenestration, and intraoperative imaging after implantation showing smooth arch branches flow. The preoperative and postoperative CTAs are shown in Figure 3. One (3.23%) patient died of sudden cerebral hemorrhage 14 days after the operation. Five patients (16.13%) developed postoperative renal insufficiency (requiring hemodialysis or urinary abnormalities or poor estimated glomerular filtration rate). Six (19.36%) patients were on a ventilator for more than 48 hours. One (3.23%) patient was found to have Type IV endoleak on undergoing CTA and there were no stent migrations.

**Table 3:** Early and the follow-up outcomes

Variable	Result
Early outcomes	
ICU stay (d)	3.00 (2.00-6.00)
Hospitalization time (d)	17.53 ± 9.94
Intubation time (h)	27.50 ± 26.55
LVEF (%)	60.21 ± 6.33
Early complication	
Stroke	0 (0.00)
Transient neurological disorder	0 (0.00)
Renal insufficiency	5 (16.13)
Cardiac insufficiency	0 (0.00)
Respiratory insufficiency	6 (19.36)
Hepatic insufficiency	3 (9.68)
Stent migration	0 (0.00)
Stent leakage, type IV	1 (3.23)
Second hemostatic surgery	0 (0.00)
In-hospital mortality	1 (3.23)
Follow-up outcomes	
Follow-up time (m)	14.00 (6.50-28.50)
Reintervention	2 (8.00)
Follow-up mortality	2 (8.00)
Follow-up stent leakage	0 (0.00)

Values are presented as n (%), median (interquartile range), or mean ± SD. *ICU*, Intensive care unit; *LVEF*, left ventricular ejection fraction.

There were 25 patients (83.33% patients) who underwent follow-up with a median follow-up time of 14.00 months (range, 6.50-28.50 months). Two (8.00%) of them died (1 of infectious shock and the other of respiratory arrest). Two (8.00%) underwent reaortic TEVAR surgery.

## Discussion

Acute aortic dissection, a catastrophic condition with mortality rates escalating by 1% to 2% hourly without intervention, mandates urgent surgical management. Untreated, mortality exceeds 50% at 48 hours and 90% by 3 months, underscoring the imperative for timely repair.^8^^,^^9^ Although open surgical repair remains the gold standard for TAAD, controversies persist regarding the optimal extent of aortic resection.^10^ Conservative strategies (ascending/hemiarch replacement) prioritize operative safety, whereas aggressive approaches (total arch replacement + frozen elephant trunk) aim to mitigate distal aortic degeneration and reintervention risks.^4^^,^^11^ In China, total arch replacement with descending aortic stenting predominates, yet concerns endure over its technical complexity, CPB, and reliance on HCA—factors exacerbating perioperative morbidity, including visceral ischemia-reperfusion injury and transfusion-associated complications (mortality ∼25%).^12^, ^13^, ^14^

Contemporary research prioritizes strategies to minimize surgical trauma and operative duration. Hybrid techniques, integrating open ascending repair with endovascular arch stabilization, have emerged to circumvent HCA and reduce systemic ischemic burden.^15^ Among these, hybrid type II protocols—avoiding circulatory arrest—demonstrate improved hemodynamic stability and lower visceral injury rates compared with conventional arch replacement.^16^ However, hybrid interventions demand dual expertise in open and endovascular techniques, necessitating meticulous procedural planning.^3^

Parallel advancements in TEVAR now enable durable management of distal aortic pathologies. Although TEVAR is established for type B dissections,^17^, ^18^, ^19^ proximal extension to the aortic arch remains challenging due to anatomical constraints. Techniques such as branched/fenestrated SGs, chimney stents, and in situ fenestration have expanded endovascular options for arch involvement.^20^, ^21^, ^22^, ^23^ However, isolated TEVAR is infeasible in TAAD due to inadequate proximal sealing zones. Thus, hybrid approaches—leveraging open ascending repair to create a stable platform for subsequent endovascular arch intervention—represent a critical innovation, balancing radical pathology correction with minimized invasiveness.^15^ This study evaluates a novel single-stage hybrid protocol combining open ascending replacement with total arch endovascular repair using intraoperatively modified fenestrated SGs, offering a tailored solution for patients with high-risk TAAD.

In summary, we consider that a hybrid 1-stage concomitant repair of the ascending aorta followed by stenting preserving the aortic arch and 3 branches of native tissue may simplify the operation and reduce the operation time.^24^^,^^25^ This technique involves intraoperative self-modification of SGs rather than relying on commercially available branched/fenestrated devices. This customization allows adaptability to individual anatomy, particularly when anatomical constraints (eg, narrow landing zones or tortuous vessels) preclude standard endovascular options. This approach has not been widely described in TAAD management and may address a gap in patients deemed unsuitable for conventional surgery or total endovascular repair. This procedure is specifically advantageous for high-risk patients with TAAD who are deemed unsuitable candidates for conventional total aortic arch replacement due to anatomical constraints, comorbidities, or physiological frailty. The key subgroups that benefit include some patients who have advantages on the dissection, patients with severe comorbidities, anatomically challenging cases with narrow aortic arch landing zones, patients requiring avoidance of HCA, TAAD with compromised distal perfusion, and reoperative scenarios. By eliminating circulatory arrest and minimizing aortic crossclamp time, our strategy may reduce neurological and systemic risks, particularly in frail patients.

This study represents an attempt to approach TAAD in a novel strategy utilizing the benefits of both interventions. Compared with traditional full open surgery, this method avoids HCA, decreases CPB times and distal body circulatory arrest, and minimizes cardiac and brain ischemia. Compared with full endovascular surgery, a proximal and safe platform is utilized for stent anchorage. Additionally, through the sternotomy approach, extra maneuvers can be utilized to guide deployment and ensure appropriate epiaortic perfusion.

At TEVAR, we close the chest only using adhesive membrane mainly to avoid reheparinization and readministration of protamine, which exacerbates the risk of thoracic hemorrhaged and failure to observe the bleeding in time. Secondly it is used to give a support point when the stent is struggling to pass through the distal anastomosis. Finally, in case of inaccurate alignment, it can be treated in a timely manner, such as establishing a bypass or needle puncture of the membrane. After ascending aortic replacement, systemic heparinization was maintained (activated clotting time, 250-300 seconds) during endovascular arch repair. Protamine was administered only after confirming successful SG deployment and hemostasis.

Although this hybrid approach demonstrates promising outcomes, it is not universally applicable to all patients with TAAD. Key limitations include anatomic constraints (eg, arch branch involvement or greater curvature tears) and technical challenges such as SG instability postfenestration or fenestration misalignment. To mitigate these risks, strict patient selection is prioritized: Eligibility requires entry tears in the descending aorta with retrograde ascending extension or localized arch lesions confined to the lesser curvature. Intraoperatively, personalized fenestration strategies—such as 1 + 2 double/triple fenestrations for dispersed branches or in situ fenestration with branch stenting for subclavian involvement—optimize SG stability and branch perfusion. Real-time imaging guidance, balloon molding, and rescue stenting address alignment or sealing issues, whereas conversion to conventional arch repair remains a last-resort bailout. Postoperative surveillance ensures early detection of complications like endoleak. In our cohort, these measures achieved 100% technical success with minimal morbidity, underscoring the protocol's viability for anatomically suitable, high-risk patients when executed with precision and contingency planning. This aligns with hybrid repair principles emphasizing adaptability over circulatory arrest-dependent strategies.

This was a retrospective study that collected clinical data from patients at a single institution. Endoluminal repair is a promising alternative, and the durability of the modified stent should be confirmed in ongoing follow-up studies. The relatively small number of individuals in this study can cause errors in the prognostic results. Our surgical experiences need to be validated in a larger cohort, more centers, and a longer follow-up period.

## Conclusions

Single-stage open ascending aortic replacement combined with the total aortic endovascular arch intervention may provide satisfactory early outcomes in TAAD. This strategy may be valuable for a subgroup of patients deemed inappropriate candidates for open classic full arch repair with acceptable outcomes.

## Conflict of Interest Statement

The authors reported no conflicts of interest.

The *Journal* policy requires editors and reviewers to disclose conflicts of interest and to decline handling or reviewing manuscripts for which they may have a conflict of interest. The editors and reviewers of this article have no conflicts of interest.
